# Functional Characterization of a Putative RNA Demethylase ALKBH6 in *Arabidopsis* Growth and Abiotic Stress Responses

**DOI:** 10.3390/ijms21186707

**Published:** 2020-09-13

**Authors:** Trinh Thi Huong, Le Nguyen Tieu Ngoc, Hunseung Kang

**Affiliations:** 1 Department of Applied Biology, College of Agriculture and Life Sciences, Chonnam National University, Gwangju 61186, Korea; trinhhuongwasi@gmail.com (T.T.H.); tieungocdhtn@gmail.com (L.N.T.N.); 2 The Western Highlands Agriculture and Forestry Science Institute, Buon Ma Thuot, DakLak 63000, Vietnam; 3 Faculty of Forestry Agriculture, Tay Nguyen University, Buon Ma Thuot, DakLak 63000, Vietnam

**Keywords:** RNA demethylation, RNA methylation, ALKBH, abiotic stress, epitranscriptomics, Arabidopsis

## Abstract

RNA methylation and demethylation, which is mediated by RNA methyltransferases (referred to as “writers”) and demethylases (referred to as “erasers”), respectively, are emerging as a key regulatory process in plant development and stress responses. Although several studies have shown that AlkB homolog (ALKBH) proteins are potential RNA demethylases, the function of most ALKBHs is yet to be determined. The *Arabidopsis thaliana* genome contains thirteen genes encoding ALKBH proteins, the functions of which are largely unknown. In this study, we characterized the function of a potential eraser protein, ALKBH6 (At4g20350), during seed germination and seedling growth in *Arabidopsis* under abiotic stresses. The seeds of T-DNA insertion *alkbh6* knockdown mutants germinated faster than the wild-type seeds under cold, salt, or abscisic acid (ABA) treatment conditions but not under dehydration stress conditions. Although no differences in seedling and root growth were observed between the *alkbh6* mutant and wild-type under normal conditions, the *alkbh6* mutant showed a much lower survival rate than the wild-type under salt, drought, or heat stress. Cotyledon greening of the *alkbh6* mutants was much higher than that of the wild-type upon ABA application. Moreover, the transcript levels of ABA signaling-related genes, including *ABI3* and *ABI4*, were down-regulated in the *alkbh6* mutant compared to wild-type plants. Importantly, the ALKBH6 protein had an ability to bind to both m^6^A-labeled and m^5^C-labeled RNAs. Collectively, these results indicate that the potential eraser ALKBH6 plays important roles in seed germination, seedling growth, and survival of *Arabidopsis* under abiotic stresses.

## 1. Introduction

RNA modifications are recently emerging as an important cellular process of epigenetic gene regulation in addition to DNA methylation and histone modifications [1,2]. Approximately 160 RNA modifications in mRNAs, tRNAs, and rRNAs have been identified to date [3,4], among which the most abundant modifications are 2′-O-ribose methylation and pseudouridilation in rRNA, 5-methylcytosine (m^5^C) and 1-methylguanidine (m^1^G) in tRNA, and N^6^-methyladenine (m^6^A) in mRNA [4,5,6,7]. These modifications affect the chemical property, charge, and hydrophobicity of bases, which influence base stacking, base pairing, and interactions of RNAs with other molecules [5,7,8,9]. RNA methylations have essential roles in ribosome structure and biogenesis, codon recognition and decoding, reading frame maintenance, and translation initiation and elongation [6,10,11,12,13,14,15].

RNA methylation is modulated by RNA methyltransferases (referred to as “writers”) and demethylases (referred to as “erasers”) that install and remove methylation marks, respectively (reviewed in [16,17,18]). These methylated marks are recognized by RNA-binding proteins, referred to as “readers” [2,16,18]. Many recent studies have demonstrated the essential roles of writer components, including MTA, MTB, VIR, HAKAI, and FIP37, and several reader proteins, including ECT2, ECT3, and ECT4, involved in mRNA m^6^A methylation and interpretation in plants (reviewed in [16,17,18,19]). Through the analysis of the mutants of these writers and erasers, it has been demonstrated that m^6^A methylation in mRNAs is crucial for plant growth and development [20,21,22,23,24,25]. By contrast, the identity, biological functions, and cellular roles of eraser proteins are largely unknown. The AlkB homolog (ALKBH) proteins, which are members of the alpha-ketoglutarate (αKG) and Fe(II)-dependent dioxygenase superfamily and can remove alkyl and methyl groups from DNAs, have been suggested to function as RNA demethylases [26,27]. Mammals have nine different ALKBH family members: ALKBH1 to ALKBH8 and the fat mass and obesity-associated (FTO) protein [28,29,30,31,32]. Among them, human ALKBH5 and FTO protein have been demonstrated to function as m^6^A demethylases, which are involved in obesity, diabetes, and hypoxia response [33,34].

In plants, the role of eraser proteins has been determined in only a few cases. Thirteen *Arabidopsis* ALKBH family members have been identified by bioinformatics analysis [30,32]. Among them, the role of ALKBH9B and ALKBH10B has been demonstrated; ALKBH10B was identified as an mRNA m^6^A eraser, influencing floral transition [35], and ALKBH9B was shown to affect methylation of alfalfa mosaic virus RNA, mediating systemic infection [36]. A recent study demonstrated that mutation of *SlALKBH2*, an RNA demethylase in tomato (*Solanum lycopersicum*), delays fruit ripening [37]. However, the role of erasers in the response of plants to abiotic stresses has yet to be determined, albeit the expression patterns of *ALKBH* genes in response to abiotic stresses have been explored [16,38,39].

Given that the function of most ALKBHs still remains unknown, it is a worthwhile endeavor to determine their roles in plant growth and stress responses. As no information about the role of ALKBH6 is known in either the plant or animal system, we aimed to determine the function of ALKBH6 in growth and abiotic stress response in *Arabidopsis*. We show that ALKBH6 plays important roles in seed germination, seedling growth, and survival of *Arabidopsis* under abiotic stresses.

## 2. Results

### 2.1. The Domain Structure, Subcellular Localization, and Stress-Responsive Expression Pattern of ALKBH6 in Arabidopsis

**Figure 1:**
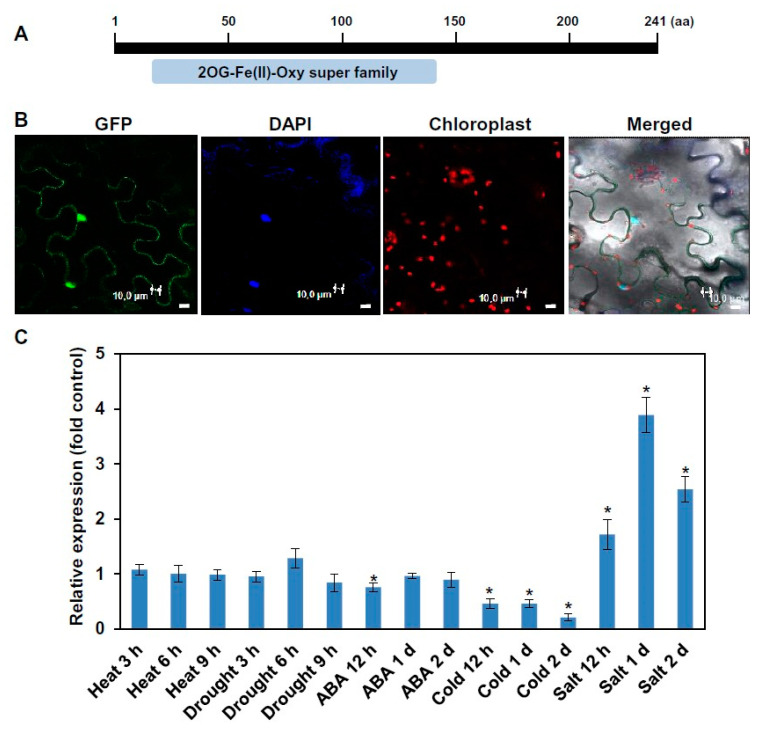
Subcellular localization and stress-responsive expression patterns of ALKBH6. (**A**) Schematic representation of the domain structure of ALKBH6. The conserved 2-oxoglutarate (2OG) and Fe(II)-dependent oxygenase (Oxy) domain is shown. aa, amino acid. (**B**) The ALKBH6-green fluorescence protein (GFP) fusion protein was transiently expressed in tobacco leaves, and the fluorescence signals were detected using a confocal microscope. 4′,6-diamidino-2-phenylindole (DAPI) was used to stain the nucleus, and the red signals indicate chloroplast auto-fluorescence. Bar = 10 μm. (**C**) Two-week-old *Arabidopsis* seedlings were subjected to drought, heat (42 °C), cold (10 °C), high salinity (300 mM NaCl), or abscisic acid (ABA) (100 μM) treatment, and the transcript levels of *ALKBH6* were determined via real-time RT-PCR. Values are the mean ± SE obtained from three independent experiments, and the asterisks above columns indicate significant differences (* *p* ≤ 0.05).

Analysis of the putative ALKB domain-containing proteins in the *Arabidopsis* genome database (http://arabidopsis.org) revealed 13 genes encoding ALKBH family members that harbor a 2-oxoglutarate-Fe(II)-Oxy-2 domain (Figure S1). ALKBH6 is a relatively small ALKBH family protein comprising 241 amino acid residues (Figure 1A). Subcellular localization of the ALKBH6 protein was investigated by transiently expressing the ALKBH6-green fluorescence protein (GFP) fusion protein in tobacco leaves. The results showed that the GFP signals were clearly merged with the 4′,6-diamidino-2-phenylindole (DAPI) signals that stain the nucleus (Figure 1B), indicating that ALKBH6 is localized in the nucleus. To obtain clues on the possible function of ALKBH6 in stress responses, the expression levels of *ALKBH6* were analyzed in *Arabidopsis* under different abiotic stresses and in the presence of abscisic acid (ABA). Successful application of the stress to the plants was verified by observing a marked increase in the transcript levels of stress marker genes, including *RD29A* for drought stress, *SUS* for salt stress, *HSP70* for heat stress, *CBF2* for cold stress, and *RD29B* for ABA (Figure S2). The expression level of *ALKBH6* was increased up to fourfold under salt stress, whereas its level was decreased by cold stress. By contrast, heat stress, drought stress, or ABA treatment did not affect *ALKBH6* expression (Figure 1C). These results suggest that ALKBH6 is involved in diverse abiotic stress responses.

### 2.2. ALKBH6 Affects Seed Germination and Seedling Growth under Abiotic Stresses

To determine the function of ALKBH6 in the growth and stress response of *Arabidopsis*, two T-DNA insertion mutants, SALK_105865C and SALK_138864C that contain the T-DNA insert in the last intron and the last exon, respectively (Figure S3A), were analyzed. Genotyping of the two mutants via genomic DNA PCR confirmed that both mutants were homozygous lines (Figure S3B). Analysis of the level of *ALKBH6* transcript in the two analyzed mutant backgrounds via RT-PCR and qRT-PCR showed that the expression of *ALKBH6* in both mutant backgrounds was approximately 10–20% of the wild-type level (Figure S3C,D). These results indicate that the two *alkbh6* mutants are knockdown mutants.

We first analyzed the rate of seed germination of wild-type plants and the *alkbh6* mutants under normal conditions, abiotic stresses, and ABA. The results showed that no differences in seed germination were observed between wild-type and *alkbh6* mutants under normal conditions (Figure S4). However, germination rates of the *alkbh6* mutants were enhanced compared to those of wild-type under salt or cold stress, as well as in the presence of ABA, but not under dehydration stress (Figure S4). These results indicate that ALKBH6 plays as a negative regulator of seed germination under salt or cold stress and in the presence of ABA.

**Figure 2:**
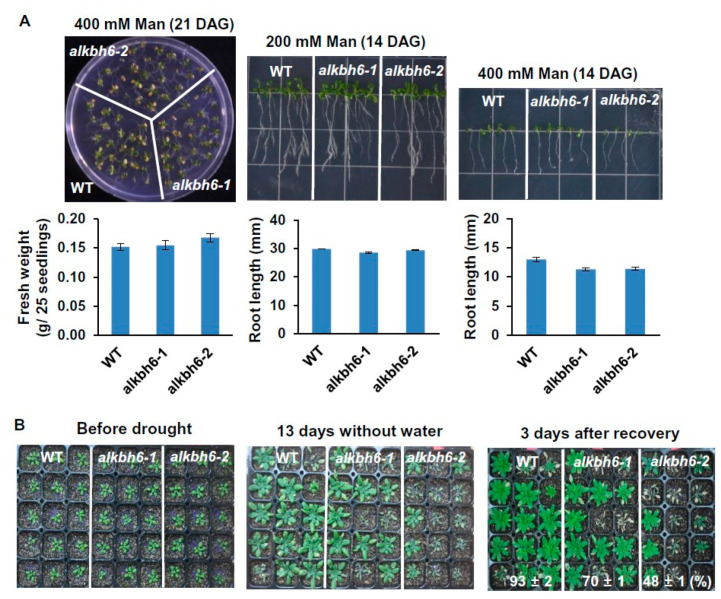
Seedling and root growth of the *alkbh6* mutants under drought stress. (**A**) The wild-type (WT) and *alkbh6* mutants were grown on the (Murashige and Skoog) MS medium supplemented with 200 or 400 mM mannitol, and the fresh weight and root length of the seedlings were measured at the indicated days after germination (DAG). (**B**) Twenty-day-old seedlings grown in soil were subjected to drought stress for 13 days, and survival rates were measured 3 days after recovery. Values are the mean ± SE obtained from three independent experiments.

We next analyzed the seedling and root growth of wild-type plants and *alkbh6* mutants under normal and abiotic stress conditions. No visible differences in overall growth were observed between wild-type plants and *alkbh6* mutants under normal growth conditions (Figure S5). When the plants were grown on solid Murashige and Skoog (MS) medium supplemented with 200–400 mM mannitol, the fresh weight and root length of the *alkbh6* mutants was similar to those of the wild-type plants (Figure 2A). However, when the 20-day-old seedlings grown in soil were subjected to drought stress by withholding watering, the survival rate of the *alkbh6* mutants was significantly lower than that of the wild-type (Figure 2B and Figure S6). These results indicate that ALKBH6 is a positive regulator of plant survival under drought stress.

**Figure 3:**
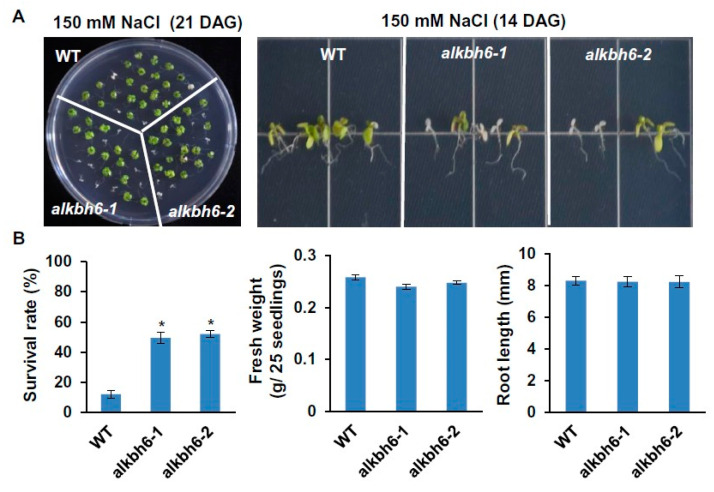
Seedling and root growth of the *alkbh6* mutants under salt stress. (**A**) Wild-type *Arabidopsis* (WT) and *alkbh6* mutants were grown on solid MS medium supplemented with 150 mM NaCl, and photographs for seedlings and roots were taken at the indicated days after germination (DAG). (**B**) Survival rate and fresh weight were measured 21 DAG, and root lengths were measured 14 DAG. Values are the mean ± SE obtained from three independent experiments, and the asterisks above columns indicate significant differences (* *p* ≤ 0.05).

**Figure 4:**
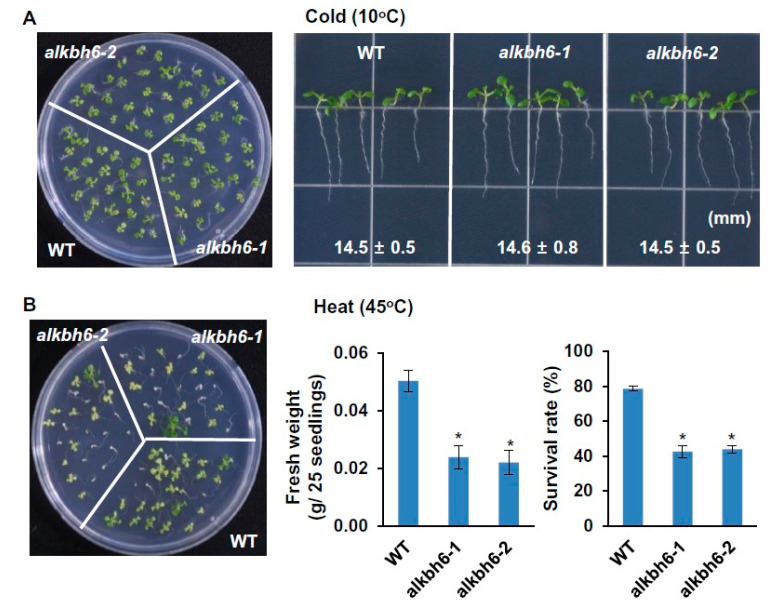
Seedling and root growth of the *alkbh6* mutants under cold or heat stress. (**A**) The wild-type (WT) and *alkbh6* mutants were grown at 10 °C, and root lengths were measured four weeks after germination. (**B**) Three-day-old seedlings were treated at 37 °C for 1 h and then subjected to heat stress at 45 °C for 4 h. Survival rate and fresh weight of the plants were measured seven days after heat treatment. Values are the mean ± SE obtained from three independent experiments, and the asterisks above columns indicate significant differences (* *p* ≤ 0.05).

Under salt stress on solid MS medium supplemented with 100–150 mM NaCl, the fresh weight and root length of the *alkbh6* mutants were similar to those of wild-type *Arabidopsis* (Figure 3A), whereas the survival rate of the *alkbh6* mutants was significantly higher than that of the wild-type (Figure 3B). No visible differences in seedling and root growth were observed between the wild-type and *alkbh6* mutants under cold stress (Figure 4A). By contrast, the seedling growth and survival rates of the *alkbh6* mutants were significantly lower than those of the wide type under heat stress; the survival rate of the wild-type and mutants after 45 °C heat treatment for 4 h were approximately 80% and 40%, respectively (Figure 4B). Collectively, these results indicate that ALKBH6 is a positive regulator of plant survival under drought or heat stress but a negative regulator of plant survival under salt stress.

### 2.3. ALKBH6 Influences the Response of Arabidopsis to ABA

**Figure 5:**
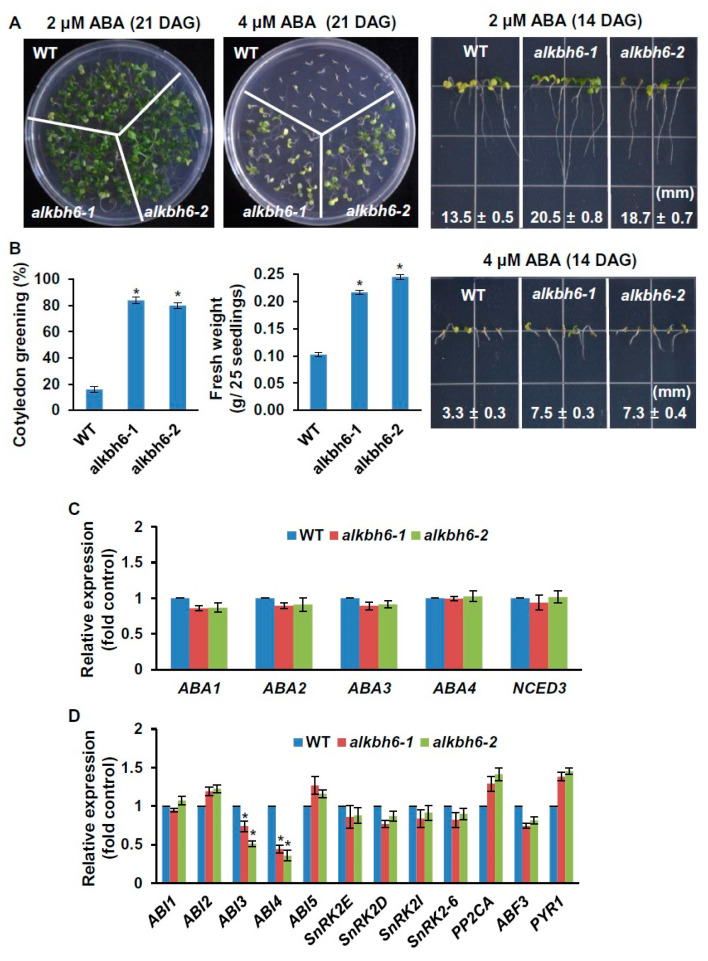
Effects of ABA on the seedling and root growth of the *alkbh6* mutants. (**A**) Growth of wild-type (WT) plants and *alkbh6* mutants was observed on solid MS medium supplemented with 2 or 4 μM ABA. (**B**) Cotyledon greening and fresh weight of the assessed plant lines were measured two and three weeks after germination, respectively. The transcript levels of (**C**) ABA biosynthesis-genes and (**D**) ABA signaling-related genes in 3-week-old seedlings were determined via qRT-PCR. Values are the mean ± SE obtained from three independent experiments, and the asterisks above columns indicate significant differences (* *p* ≤ 0.05).

To determine whether ALKBH6 plays a role in ABA response, the growth of seedlings and roots of the plant lines was analyzed in the presence of different concentrations of ABA. When the wild-type and *alkbh6* mutants were grown on solid MS medium supplemented with 2 or 4 μM ABA, the fresh weight, cotyledon greening, and root lengths of the *alkbh6* mutants were significantly elevated compared to corresponding measurements determined for wild-type *Arabidopsis* (Figure 5A,B). To ascertain how ALKBH6 affects the seedling growth of *Arabidopsis* in the presence of ABA, the expression levels of ABA biosynthesis- and ABA signaling-related genes were analyzed via qRT-PCR in the wild-type and *alkbh6* mutants upon ABA application. The results showed that the levels of the ABA biosynthesis-related genes, including *ABA1, ABA2, ABA3, ABA4*, and *NCED3*, were not altered in the *alkbh6* mutants upon ABA application (Figure 5C), whereas the levels of the ABA signaling-related genes, including *ABI3* and *ABI4*, were significantly decreased in the mutants compared to those in the wild-type (Figure 5D). These results indicate that ALKBH6 affects ABA response via modulation of the expression of ABA signaling-related genes.

### 2.4. ALKBH6 Binds to the RNA with m^6^A or m^5^C Modification

The next important question we aimed to answer was, what is the RNA target of ALKBH6? Because no information is currently available for the potential RNA target of ALKBH6, we first analyzed whether the m^6^A or m^5^C level was altered in the *alkbh6* mutants using the commercially available m^6^A and m^5^C assay kits. The results showed no significant differences in m^6^A and m^5^C levels between the wild-type and *alkbh6* mutants (Figure S7).

**Figure 6:**
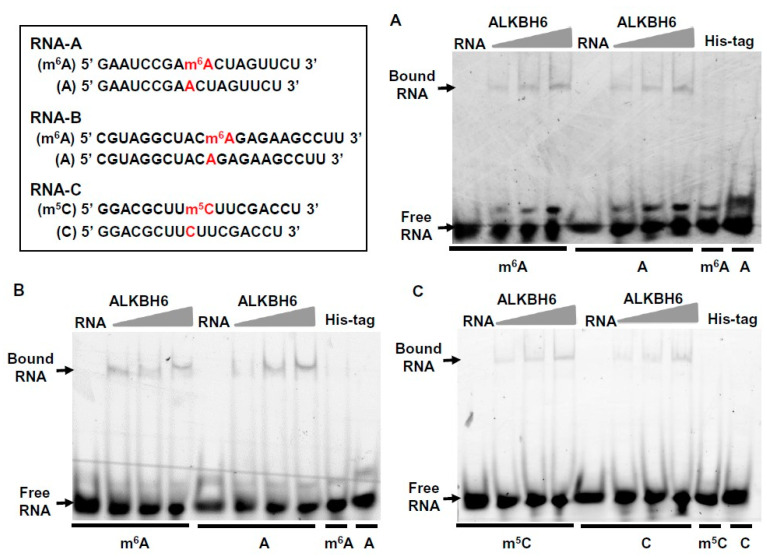
RNA-binding ability of the ALKBH6 protein. Electrophoretic mobility shift assay showing the binding between the ALKBH6 protein (3, 5, and 7 μM) and the synthetic (**A**) RNA-A, (**B**) RNA-B, or (**C**) RNA-C. The modified adenine and cytosine bases are indicated in red. The 5′-fluorescein-labeled RNA substrates were mixed with the purified protein, and the reaction mixtures were separated on a 6% native acrylamide gel. The binding products were detected using an image analyzer.

Although the knockdown of the expression level of ALKBH6 did not alter the m^6^A- and m^5^C-level, we next evaluated whether ALKBH6 can bind to an m^6^A- or m^5^C-modified RNA. The recombinant ALKBH6 protein was purified, which contained ALKBH6 and a small amount of His-tag-Trigger factor present in the pCold trigger factor (TF) DNA vector (Figure S8). The 5′-fluorescein-labeled RNA substrates were synthesized and used in an electrophoretic mobility shift assay (EMSA). Two m^6^A-labeled RNA substrates were used in this assay; one RNA substrate containing a conserved RRACH motif (R = G/A, H = U/A/C), which was derived from the *Arabidopsis* Salt Overly Sensitive 3 (SOS3) RNA (5′-GAAUCCGAm^6^ACUAGUUCU-3′ and 5′-GAAUCCGAACUAGUUCU-3′) [40], and another RNA substrate containing a conserved UACAGAGA motif, which was derived from the human S-adenosylmethionine synthase (MAT2A) RNA (5′-CGUAGGCUACm^6^AGAGAAGCCUU-3′ and 5′-CGUAGGCUACAGAGAAGCCUU-3′) [41]. The m^5^C-labeled RNA substrate was derived from the conserved m^5^C motif found in *Arabidopsis* (5′-GGACGCUUm^5^CUUCGACCU-3′ and 5′-GGACGCUUCUUCGACCU-3′) [42]. The results of the EMSA showed that the addition of ALKBH6 retarded the migration of both m^6^A-labeled- and unlabeled-RNAs, as well as both m^5^C-labeled- and unlabeled-RNAs (Figure 6). By contrast, the addition of His-tag-Trigger factor alone did not retard the migration of the RNAs. Collectively, these results indicate that ALKBH6 has an ability to bind to m^6^A- or m^5^C-labeled RNAs, suggesting its possible role as an m^6^A or m^5^C eraser.

## 3. Discussion

RNA demethylation mediated by eraser proteins is emerging as an important cellular process of epigenetic gene regulation in the response of plants to abiotic stresses, as well as in plant development. In particular, the potential role of ALKBHs in stress response has been suggested based on their altered expression patterns upon stress treatment [16,38,39,43]. However, the function of most of the potential ALKBH eraser proteins remains unknown. The results presented in this study demonstrate that ALKBH6, one of the 13 AlkB domain-containing proteins encoded by *Arabidopsis* (Figure S1), plays a role in seed germination and post-germination seedling growth of *Arabidopsis* under abiotic stresses. Although no differences in seed germination and seedling growth were observed between wild-type *Arabidopsis* and the *alkbh6* mutants under normal conditions (Figures S4 and S5), the *alkbh6* knockdown mutants germinated faster than wild-type plants under cold or salt stress, but not under dehydration stress (Figure S4). These results indicate that ALKBH6 negatively influences seed germination under various abiotic stresses. Evidently, the *alkbh6* mutants showed a lower survival rate than wild-type plants under drought or heat stress, but a higher survival rate than the wild-type under salt stress (Figure 2, Figure 3 and Figure 4), indicating that ALKBH6 mediates different roles in the *Arabidopsis* response to various abiotic stresses. The importance of RNA methylation in stress responses in plants has been demonstrated in only a few cases; OsNUSN2, an mRNA m^5^C methyltransferase in rice, was shown to regulate mRNA translation and maintain chloroplast function, which enhances rice adaptation to heat stress [44], and m^6^A methylation was associated with the stabilization of the transcripts encoding salt response proteins in response to salt stress [45]. Although we do not know the mechanistic role of ALKBH6 in stress responses, our current results together with these previous findings point to the crucial role of RNA methylation/demethylation in the response of plants to abiotic stresses.

Notably, the *alkbh6* mutants were hyposensitive to ABA that inhibits seed germination and the post-germination growth of plants [46]; the seed germination, cotyledon greening, and seedling growth of the *alkbh6* mutants were all elevated compared to these metrics in wild-type plants in the presence of ABA (Figure 5 and Figure S4). The expression levels of ABA signaling-related genes, including *ABI3* and *ABI4,* were significantly decreased in the mutant backgrounds compared to their levels in wild-type plants upon ABA application (Figure 5D). As *ABI3* and *ABI4* are known to inhibit seed germination and seedling growth of *Arabidopsis* via ABA signaling [47,48,49,50,51,52], these results indicate that ALKBH6 acts as a negative regulator of the ABA response via influencing ABA signaling. Although we do not know presently how the transcript levels of *ABI3* and *ABI4* are decreased in the *alkbh6* mutants, a possible clue can be obtained from the observation that RNA methylation is associated with the stability of mRNAs; m^6^A methylation was shown to either decrease or increase the stability of mRNAs in animals and plants [37,45,53,54,55]. It is, therefore, likely that ALKBH6-mediated demethylation influences the stability of RNA targets. Further studies are needed to determine whether ALKBH6 either directly or indirectly regulates the levels of *ABI3* and *ABI4* during ABA response.

Finding RNA targets of an RNA demethylase is crucial for understanding the cellular and mechanistic role of the eraser protein. Previous studies have identified many base methylations in tRNAs [56], rRNAs [57], and mRNAs [4]. In particular, based on a recent advance in transcriptome-wide methylated RNA immunoprecipitation-sequencing (MeRIP-seq), m^6^A was found to be the most abundant internal modification in eukaryotic mRNAs [2,17]. Three conserved motifs for m^6^A methylation have been identified to date; the RRACH motif (R = G/A, H = U/A/C) in plants and animals [35,40,53,58], the UG(U/A)A(C/A)H motif in plants [37,59], and the ACAGAGA motif in humans [41,60]. Although the writer proteins that install these m^6^A marks have been identified, the eraser proteins targeting only the RRACH motif have been identified, including the human ALKBH5 and FTO proteins [33,34] and plant ALKBH9B and ALKBH10B proteins [35,36]. The function and RNA target of ALKBH6 are unknown in plants as well as in animals [32]. Previous studies have demonstrated that ALKBH6 in humans is localized mainly in the nucleus and also in cytoplasm [30,61], and that ALKBH6 possesses a positively charged surface, suggesting its possible interaction with nucleic acids [62]. Given that the potential RNA target of ALKBH6 is elusive, it will be of worth to evaluate the RNA-binding ability of ALKBH6. Our results clearly show that ALKBH6 has an ability to bind to both m^6^A-labeled and m^5^C-labeled RNAs (Figure 6), raising the possibility that it may be an m^6^A or m^5^C eraser. Contrary to this expectation, our results showed that m^6^A and m^5^C levels are not significantly altered in the *alkbh6* mutants (Figure S6). The lack of reduced levels of m^6^A or m^6^C in the mutants is possibly due to the residual demethylase activity in the *alkbh6* knockdown mutants or the limited sensitivity of the commercially available m^6^A and m^5^C detection kits used in this study. Further analyses of the m^6^A or m^6^C level in the loss-of-function *alkbh6* knockout mutant and via using a more sensitive RNA methylation analysis method, such as LC-MS/MS, are needed to prove this possibility. Given that ALKBH6 is localized in the nucleus (Figure 1), it is likely that ALKBH6 is involved in the maintenance of proper levels of RNA methylation in the nucleus, which affects the processing or stability of target RNAs. The next critical experiments will be to characterize the demethylase activity of ALKBH6 and to determine whether ALKBH6 targets mRNA, rRNA, or tRNA. Owing to recent advances in novel technologies for detecting and mapping RNA modifications, including MeRIP-seq, m^6^A-CLIP/miCLIP, m^6^A-LAIC-seq, and PA-m^6^A-seq [17], it is practically feasible, albeit not easy, to identify RNA targets of an eraser protein. An additional future major task is to utilize these technologies to map the transcriptome-wide methylation status of RNAs in the *alkbh6* mutant background and in other eraser mutant backgrounds, which will provide crucial information to furthering our understanding of the cellular roles of ALKBH eraser proteins in plants.

## 4. Materials and Methods

### 4.1. Plant Materials and Growth Conditions

*A. thaliana* Columbia-0 (Col-0) ecotype was used in this study. Two *Arabidopsis alkbh6* T-DNA insertion mutants (SALK_105865C and SALK_138864C) were obtained from the Arabidopsis Biological Resource Center (Columbus, OH, USA). The seeds were sterilized using 70% ethanol and 2% NaClO, and then washed with sterile water six times. The seeds were sown on half-strength Murashige and Skoog (MS) (Duchefa Biochemie, Haarlem, The Netherlands) medium containing 1% sucrose and were maintained at 4 °C for 3 days in darkness. The plants were grown in a growth room maintained at 23 ± 2 °C under long-day conditions (16 h light/8 h dark cycle).

### 4.2. Germination and Seedling Growth Assays under Abiotic Stresses

The wild-type and mutant seeds were harvested at the same time and were used for the analysis of germination and seedling growth, as described previously [63]. In short, to evaluate the effects of salt stress, drought stress, or ABA treatment on seed germination and seedling growth, the seeds were sown on solid MS medium supplemented with 100–250 mM NaCl, 200–400 mM mannitol, or 1–4 μM ABA, respectively. For cold stress treatment, the MS plates containing the seeds were placed at 10 °C under long-day conditions. For heat stress treatment, 3-day-old seedlings were treated at 37 °C for 1 h and then subjected to heat stress at 45 °C for 4 h. Survival rate and fresh weight of the plants were measured seven days after heat treatment. Each seed was regarded as germinated when the radicle emerged from the seed coat. The fresh weight, root length, or survival rates of the seedlings were measured at different time points. To evaluate the effects of abiotic stress or ABA on root growth, the seeds were sown on the MS plates supplemented with NaCl, mannitol, or ABA, and the plates were placed in a vertical orientation. For drought stress treatment in soil, 6-day-old seedlings grown on MS medium were transferred to soil and were further grown for 14 days. Drought stress was applied by stopping watering for 13 days, and the recovery rate of each genotype was scored 3 days after re-watering. All experiments were repeated at least three times.

### 4.3. Analysis of Subcellular Localization of the ALKBH6 Protein

To generate the ALKBH6-green fluorescence protein (GFP) fusion construct, the cDNA encoding the full-length ALKBH6 was fused in front of the GFP gene using the *Bam*HI/*Xba*I site in the CsV-GFP3-PA vector. The resulting plasmid was transformed into *Agrobacterium tumefaciens* GV3401, and the ALKBH6-GFP fusion protein was transiently expressed in tobacco leaves via *Agrobacterium* infiltration. The GFP signals emerging from the ALKBH6-GFP protein were observed using a confocal microscope (Carl Zeiss, Inc., Thornwood, NY, USA) with the excitation and emission wavelengths of 488 and 505–545 nm, respectively.

### 4.4. RNA Extraction and Real-Time RT-PCR

The plant samples collected at different time points were quickly frozen and ground to fine powder using a mortar and pestle in liquid nitrogen. Total RNA was extracted using the TRIzol reagent (GenAll^®^ Hybrid-R^TM^, Seoul, Korea), and the concentration of RNAs was qualified using a NanoDrop 2000 spectrophotometer (Thermo Scientific, Wilmington, DE, USA). To examine the level of gene expression, two hundred nanograms of total RNAs was used for RT-PCR and real-time qRT-PCR using a Rotor-Gene Q real-time thermal cycling system (Qiagen, Valencia, CA, USA) and QuantiTest SYBR Green RT-PCR kit (Qiagen) using the primers listed in Table S1. All experiments were repeated at least three times.

### 4.5. Vector Construction and the Expression of Recombinant Protein in Escherichia coli

To express the ALKBH6-His-taged fusion protein, the full-length cDNA encoding *ALKBH6* was cloned into the pCold TF DNA vector (Takara, Shiga, Japan) using the *Bam*HI/*Sal*I sites. The resulting plasmid was transformed into the *E. coli* BL21 cell. After culturing the cells at 37 °C until OD_600_ reached 0.4–0.8, the cultures were quickly cooled to 15 °C in ice water for 30 min, after which the synthesis of the recombinant protein was induced by adding 0.1 mM IPTG at 15 °C. The *E. coli* cells were collected, re-suspended in 1.0% PBS, pH 7.4, and disintegrated by sonication. The ALKBH6-His-taged fusion protein was digested with the HRV 3C protease (Takara), and the ALKBH6 protein was purified using a Ni-NTA His•Bind resin (Novagen, Temecula, CA, USA) using the elution buffer containing 250–500 mM imidazole. The total and purified proteins were analyzed via SDS-12% PAGE.

### 4.6. RNA-Protein Binding Assay

RNA-protein binding assay was conducted as described previously [64]. In short, the 5′-fluorescein-labeled RNA substrates with and without m^6^A or m^5^C methylation were synthesized (BIONEER, Seoul, Korea). Approximately 3–7 μM of the purified recombinant protein was mixed with 5.0 pmol of the synthetic RNA in a 20 μL binding buffer (10 mM Tris-HCl, pH 8.0, 150 mM NaCl, 1.0 mM EDTA, and 10% glycerol) on ice for 30 min. The RNA-protein mixture was loaded on a 6% polyacrylamide gel in 0.25 × Tris/Borate/EDTA (TBE) at 4 °C, and the binding products were detected using a FLA7000 image analyzer (GE Healthcare Life Sciences, Uppsala, Sweden).

### 4.7. Measurement of the m^6^A/A and m^5^C levels

Total RNA in seedlings was extracted using a Plant RNeasy extraction kit (Qiagen) following the manufacturer’s instructions. The level of m^6^A was determined using an EpiQuik m^6^A RNA Methylation Quantification Kit (EPIGENTEK, Farmingdale, NY, USA), and the level of m^5^C was determined using a MethylFlash 5-mC RNA Methylation ELISA Easy Kit (EPIGENTEK) according to the manufacturer’s instructions. In brief, 200 ng of RNA was bound to the assay wells containing an m^6^A- or m^5^C-specific antibody. The capture and detection antibody solutions were added, the signals for m^6^A were quantified colorimetrically by reading the absorbance at 450 nm, and the signals for m^5^C were quantified by measuring the fluorescence at the excitation and emission wavelengths of 530 and 590 nm, respectively. The m^6^A and m^5^C levels were calculated based on the constructed standard curves.

## Supplementary Materials

Supplementary materials can be found at https://www.mdpi.com/1422-0067/21/18/6707/s1. Figure S1: Domain structures of the *ALKB* domain-containing proteins in *Arabidopsis*; Figure S2: The expression patterns of the stress-responsive marker genes in *Arabidopsis*; Figure S3: Confirmation of the T-DNA insertion *alkbh6* mutants; Figure S4: Seed germination of the *alkbh6* mutants under normal and abiotic stress conditions; Figure S5: Phenotypes of the *alkbh6* mutants under normal growth conditions; Figure S6: Drought sensitivity of the *alkbh6* mutants; Figure S7: Levels of m6A/A and m5C in the *alkbh6* mutants; Figure S8: Purification of the recombinant ALKBH6 protein; Table S1: Gene-specific primer pairs used in this study.

## Abbreviations

ALKBH	AlkB homolog
ABA	Abscisic acid
GFP	Green fluorescent protein
