# Supplementary material for: Functional Characterization of a Putative RNA Demethylase ALKBH6 in Arabidopsis Growth and Abiotic Stress Responses
Source: Int J Mol Sci. 2020 Sep 13;21(18):6707. doi: 10.3390/ijms21186707 (PMC7555452; doi:10.3390/ijms21186707)
Supplement: Supplementary file 1 [file ijms-21-06707-s001.pdf]

Table S1. Gene-specific primer pairs used in this study

Gene	Primers (5' to 3')
**For protein expression construct**	
ALKBH6	Forward: GGATCCATGGAGTTAGAGAGATTCAGG<br>Reverse: GTCGACGAATCTGAAGAGATTTTTACG
**For genomic DNA PCR**	
ALKBH6	Forward: TCATTCTCTGTCTTGTTGATGCC<br>Reverse: CCAAAGCTTCAGCTTCATTAACG<br>Forward: TTCCTCCACGGCATTAGCGATAG<br>Reverse: ACGGACTTTGGGAACTAGACGGC
**For RT-PCR**	
ALKBH6	Forward: TCAAGATGGACCCGCTTACTTTCC<br>Reverse: ACGGACTTTGGGAACTAGACGGC
**For quantitative real-time RT-PCR**	
ALKBH6	Forward: TGTCTCTTGGCTCACCTGTTGT<br>Reverse: ACGGACTTTGGGAACTAGACGGC
RD29A	Forward: CGGAGGAAATTATTCCACCAGGGA<br>Reverse: ACAGAATGAGCCGGTGCATCGTG
RD29B	Forward: TCCTGTCGTGTCTTCTGACCACAC<br>Reverse: TCGCTTCCCAGTCCGATGTTTCC
CBF2	Forward: GGTCTTGACATGGAGGAGGAGACC<br>Reverse: CAACATCGCCTCTTCATCCATA
SUS	Forward: TAAGCTCTTCCATGACAAGGAGA<br>Reverse: AAGGTGTGTTGCAGAGTGTTGA
HSP70	Forward: GGGCACGAACAAAGGACAAC<br>Reverse: GCCCAGCTCCTTGGTACATT
ABA1	Forward: GAACGTACTATAAAGGGAGAAGG<br>Reverse: CTGAGACGAAGGGATCACAAT
ABA2	Forward: CTAAACTCGCTTTGGCTCATT<br>Reverse: CGCTACATCATCAACCGTCAG
ABA3	Forward: TTTGCTGGGATGACAATGAT<br>Reverse: CCTTCCACTGACGACGGTTC
NCED3	Forward: TAAAAACCGTTGGTCGGTTC<br>Reverse: CGACGTCCGGTGATTTAGTT
ABI1	Forward: AGAGTGTGCCTTTGTATGGTTTA<br>Reverse: CATCCTCTCTCTCTACAATAGTTCGCT
ABI5	Forward: CAATAAGAGAGGGATAGCGAACGAG<br>Reverse: CGTCCATTGCTGTCTCCTCCA
SnRK2D	Forward: TTGTTATGGAATATGCTGCTGGTGG<br>Reverse: TCAGATCCCGATGGCATATTTGC
SnRK2E	Forward: AACTCCTGCTTAATCGCTCCTAGAG<br>Reverse: TTTCCTGAAATTCTTTGGTCCTCG
SnRK2I	Forward: GCAATGCAGGACGGTTTAGTGAAG<br>Reverse: TTAATCGAGGAGCAGGACTTCCATC
SnRK2-6	Forward: TTTGTTGCTGACCCTGCAAAGAGG<br>Reverse: TCTTCTATGCTTTGGCCCGGTTGA
ABF3	Forward: GGTTGATGATGTCTGGAAGGAGC<br>Reverse: CCCTAACCACACCAGCCCTGA
PYR1	Forward: GCGAACACATCAACGGAAAG<br>Reverse: CCAGATCCGATTCTCTTTCTCG

Supplementary Figure S1. Domain structures of the *ALKB* domain-containing proteins in *Arabidopsis.* Full-length amino acid sequences of the thirteen ALKB domain-containing proteins were downloaded from the *Arabidopsis* genome sequence database (http://arabidopsis.org) and analyzed using a SMART program (http://smart.embl.de). Blue bars represent the conserved 2-oxoglutarate (2OG) and Fe(II)-dependent oxygenase (Oxy) domains.

Supplementary Figure S2. The expression patterns of the stress-responsive marker genes in *Arabidopsis*. Two-weeks-old *Arabidopsis* Col-0 seedlings were subjected to drought, heat (42°C), cold (10°C), salt (300 mM NaCl) stress, or ABA treatment (100 µM ABA) for the indicated times, and the expression levels of each gene were determined via quantitative real-time RT-PCR. Values are the mean ± SE obtained from three independent experiments.

Supplementary Figure S3. Confirmation of the T-DNA insertion *alkbh6* mutants. (A) Schematic representation of the T-DNA insertion site. Exons and introns are represented by black boxes and lines, respectively. The position of T-DNA insertion in the mutant is indicated by a black triangle. (B) Genomic DNA PCR analysis to confirm the homozygous line of the mutant. (C) RT-PCR analysis to show the down regulation of *ALKBH6* expression in the mutants. Tubulin was used as a loading control. (D) Real-time RT-PCR analysis to confirm the reduced level of *ALKBH6* in the mutants. Values are the mean ± SE obtained from three independent experiments.

Supplementary Figure S4. Seed germination of the *alkbh6* mutants under normal and abiotic stress conditions. Germination percentages of the wild type and *alkbh6* mutants were scored on the MS medium supplemented with different concentrations of mannitol, NaCl, or ABA, and on MS medium at cold temperature (10℃). The mean values and standard errors (bar) were obtained from three independent experiments.

Supplementary Figure S5. Phenotypes of the *alkbh6* mutants under normal growth conditions. Growth of the wild type and *alkbh6* mutants (A) on MS and (B, C) in soil under normal growth conditions. DAG, days after germination.

Supplemental Figure S6. Drought sensitivity of the *alkbh6* mutants. Twenty-days-old seedlings grown in soil were subjected to drought stress for 13 days, and survival rates were measured 3 days after recovery.

Supplementary Figure S7. Levels of m^{6}A/A and m^{5}C in the *alkbh6* mutants. Total RNA in 18-days-old wild type (WT) and *alkbh6* mutant (6-1 and 6-2) seedlings was extracted, and the levels of m^{6}A/A (%) and m^{5}C (%) were determined using an EpiQuik m^{6}A RNA Methylation Quantification Kit and a MethylFlash 5-mC RNA Methylation ELISA Easy Kit, respectively. Values are the mean ± SE obtained from three independent experiments. NC, negative control; PC, positive control.

Supplementary Figure S8. Purification of the recombinant ALKBH6 protein. The His-tag-ALKBH6 fusion protein was expressed in *E. coli* and purified using a Ni-NTA His•Bind resin. M, molecular size marker; U, un-induced; I, induced; P, purified.

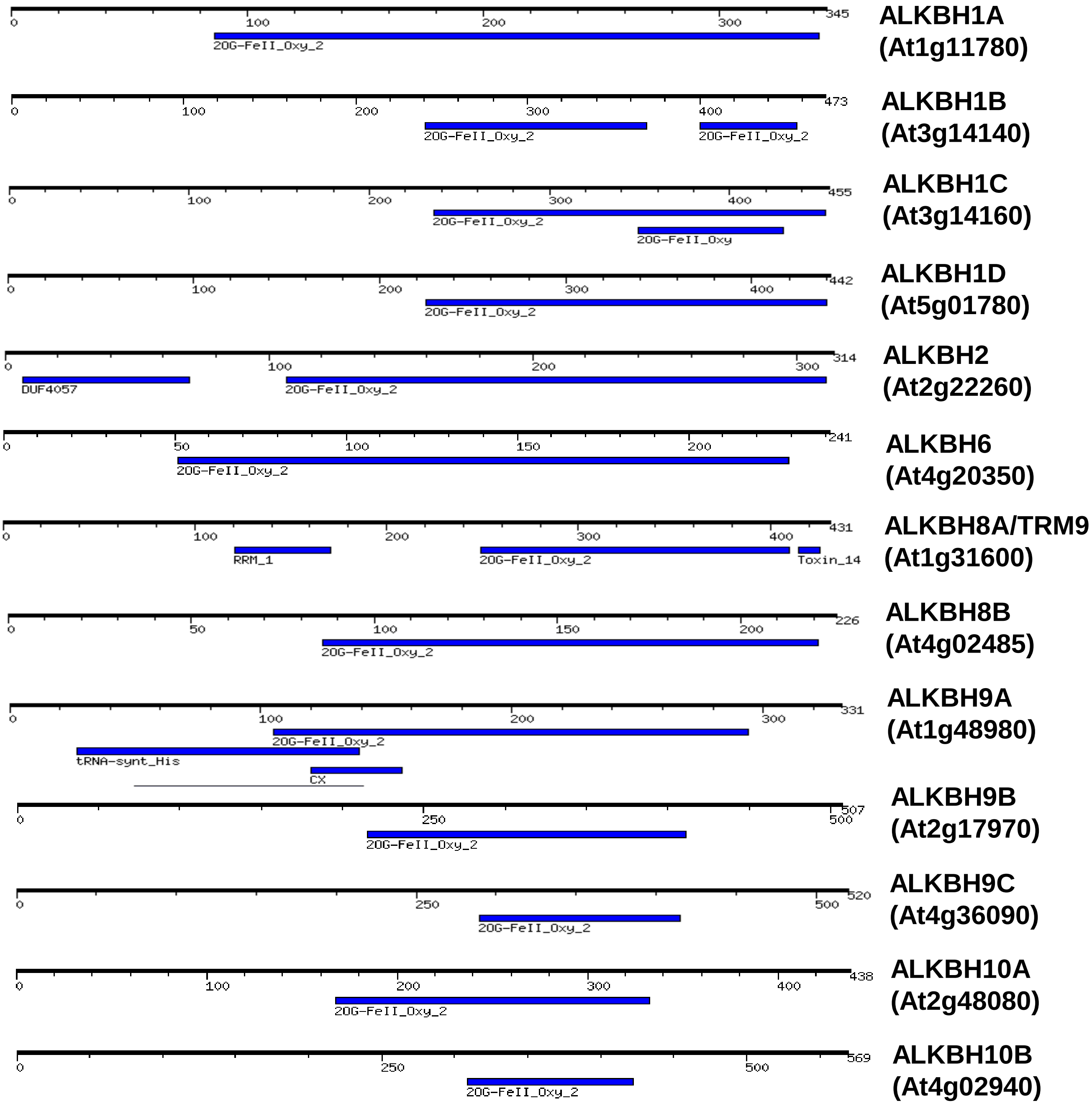

Supplementary Figure S1. Domain structures of the ALKB domain-containing proteins in *Arabidopsis*. Full-length amino acid sequences of the thirteen ALKB domain-containing proteins were downloaded from the *Arabidopsis* genome sequence database (http://arabidopsis.org) and analyzed using a SMART program (http://smart.embl.de). Blue bars represent the conserved 2-oxoglutarate (2OG) and Fe(II)-dependent oxygenase (Oxy) domains..

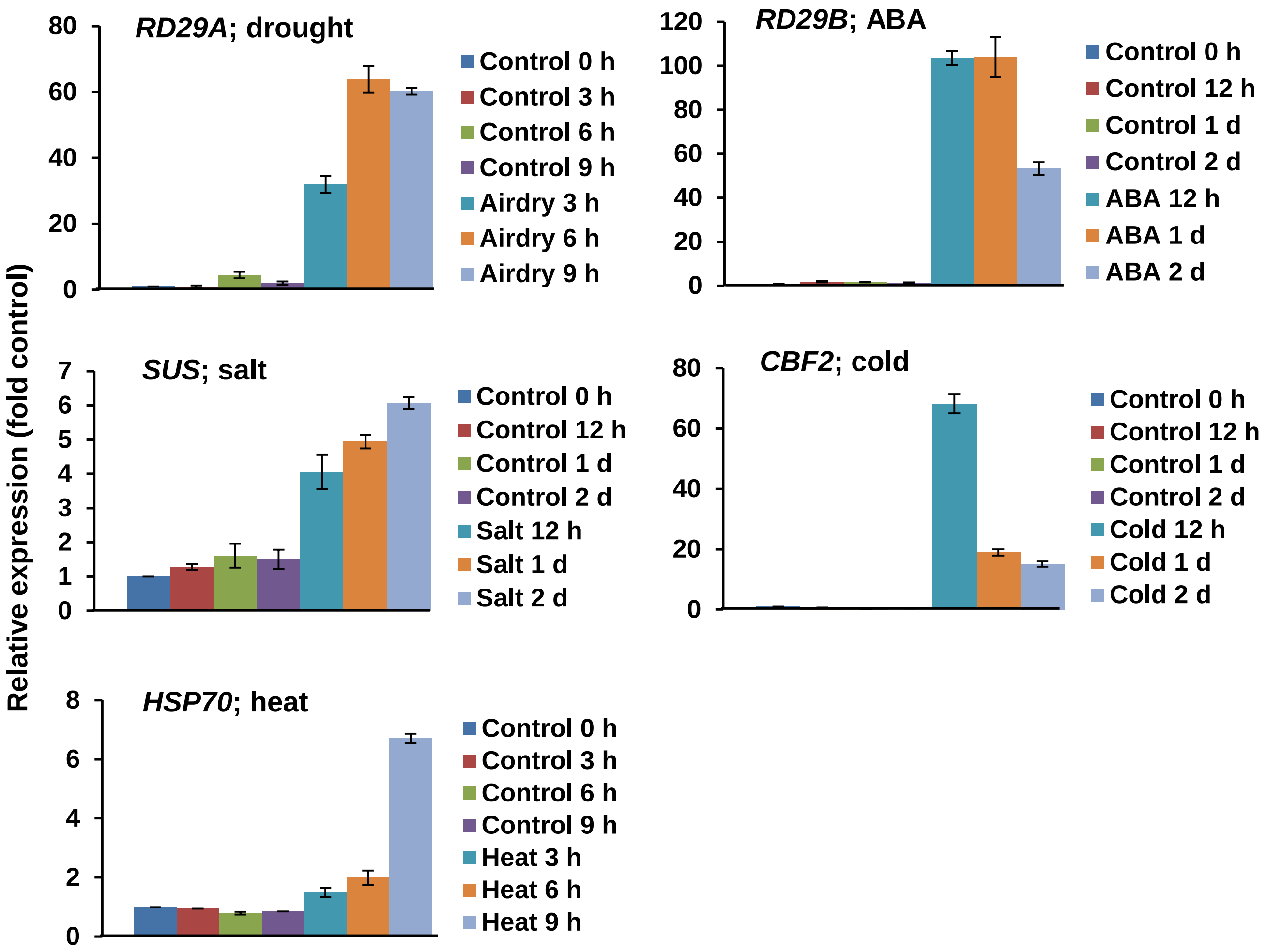

Supplementary Figure S2. The expression patterns of the stress-responsive marker genes in *Arabidopsis*. Two-weeks-old *Arabidopsis* Col-0 seedlings were subjected to drought, heat (42°C), cold (10°C), salt (300 mM NaCl) stress, or ABA treatment (100 µM ABA) for the indicated times, and the expression levels of each gene were determined via quantitative real-time RT-PCR. Values are the mean ± SE obtained from three independent experiments.

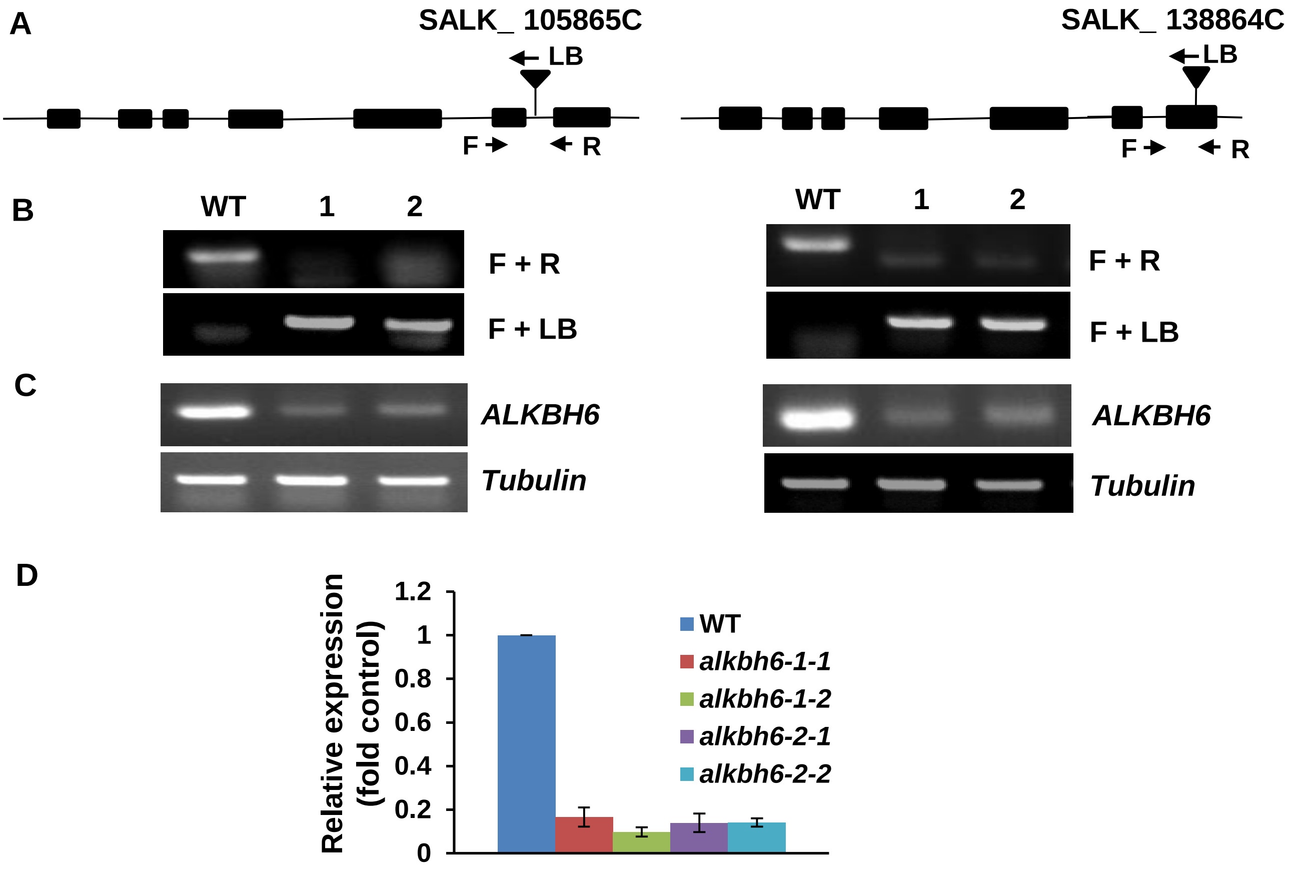

Supplementary Figure S3. Confirmation of the T-DNA insertion *alkbh6* mutants. (A) Schematic representation of the T-DNA insertion site. Exons and introns are represented by black boxes and lines, respectively. The position of T-DNA insertion in the mutant is indicated by a black triangle. (B) Genomic DNA PCR analysis to confirm the homozygous line of the mutant. (C) RT-PCR analysis to show the down regulation of *ALKBH6* expression in the mutants. Tubulin was used as a loading control. (D) Real-time RT-PCR analysis to confirm the reduced level of *ALKBH6* in the mutants. Values are the mean ± SE obtained from three independent experiments.

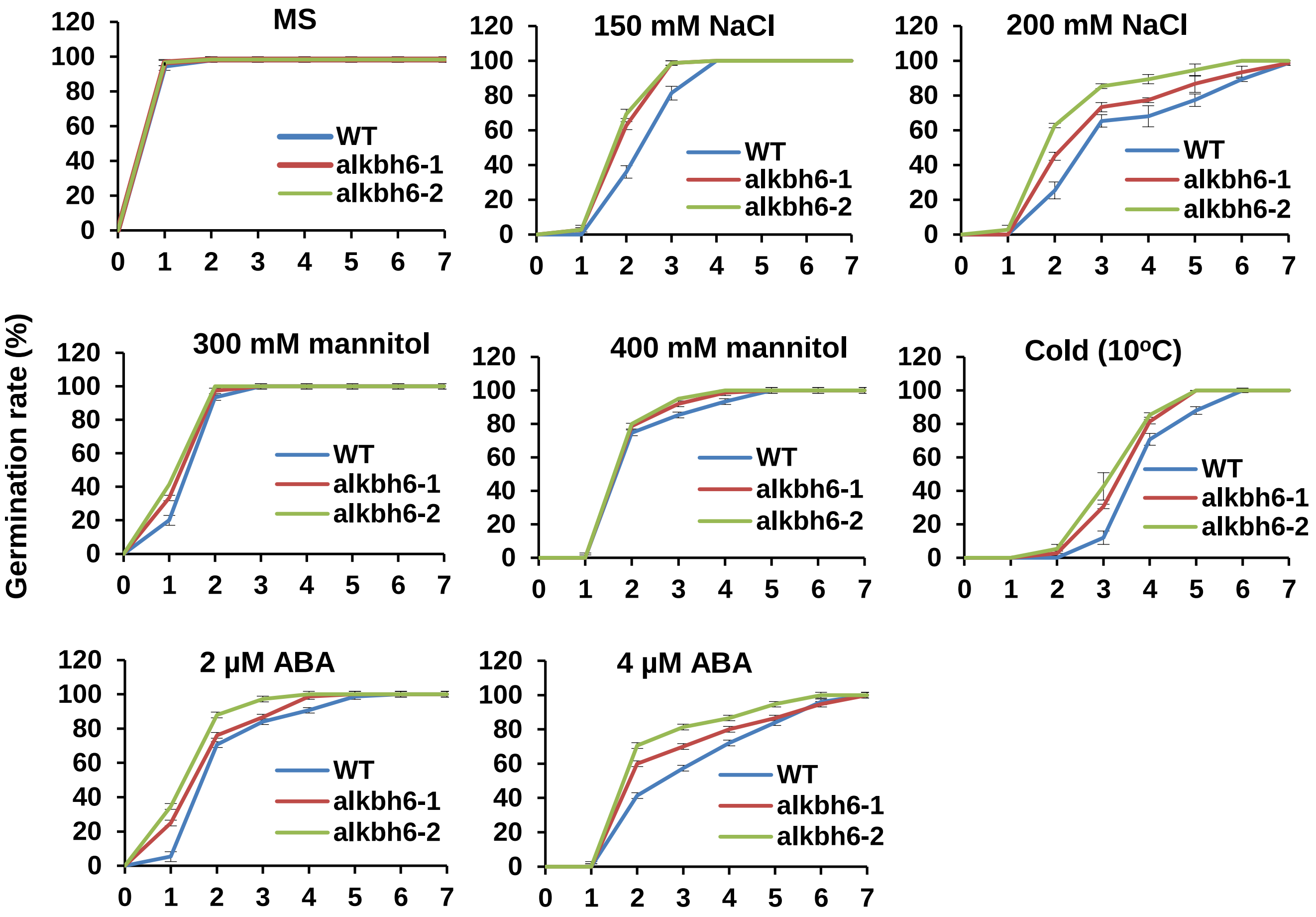

Supplementary Figure S4. Seed germination of the *alkbh6* mutants under normal and abiotic stress conditions. Germination percentages of the wild type and *alkbh6* mutants were scored on the MS medium supplemented with different concentrations of mannitol, NaCl, or ABA, and on MS medium at cold temperature (10ºC). The mean values and standard errors (bar) were obtained from three independent experiments.

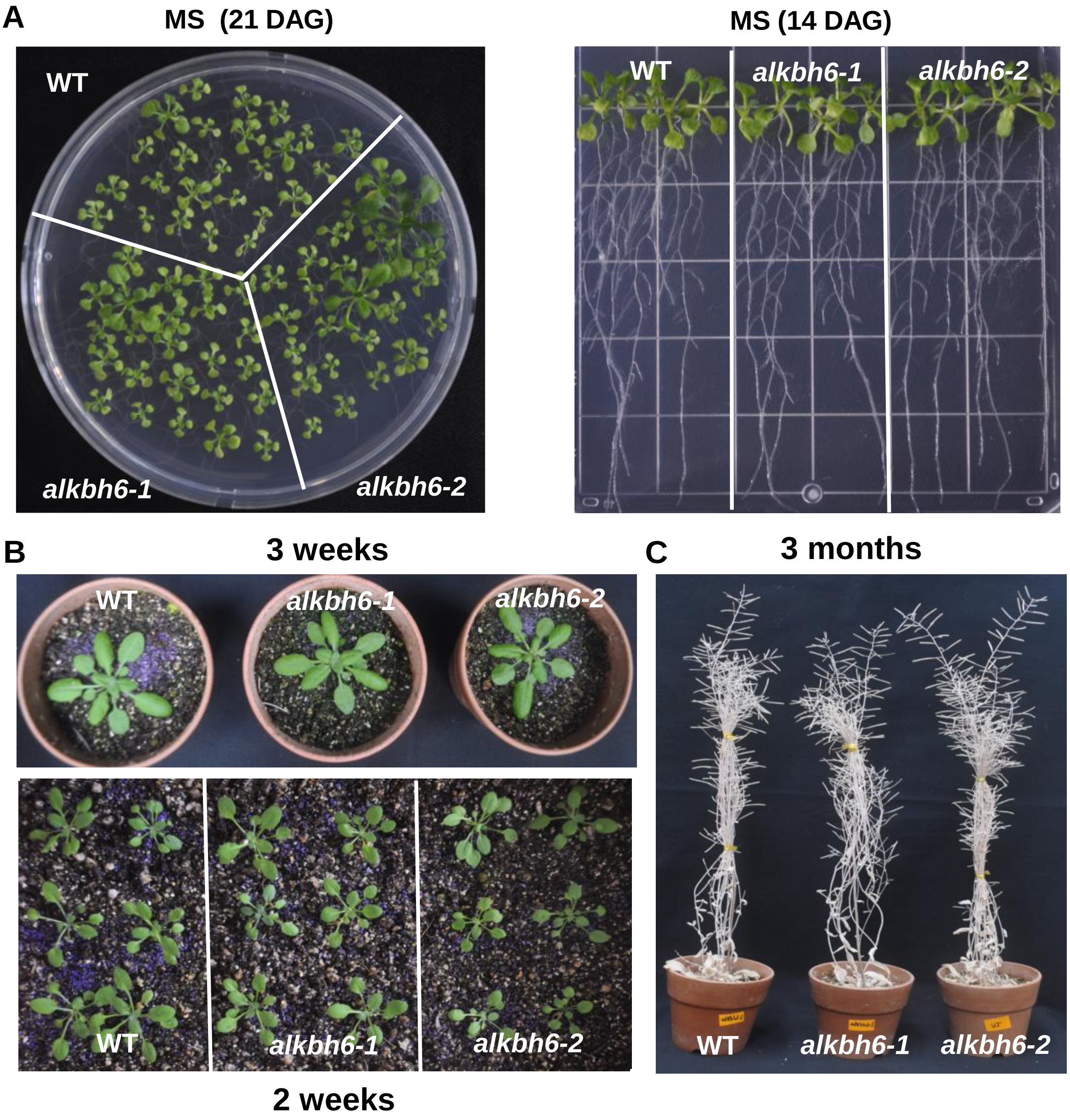

Supplementary Figure S5. Phenotypes of the *alkbh6* mutants under normal growth conditions. Growth of the wild type and *alkbh6* mutants (A) on MS and (B, C) in soil under normal growth conditions. DAG, days after germination.

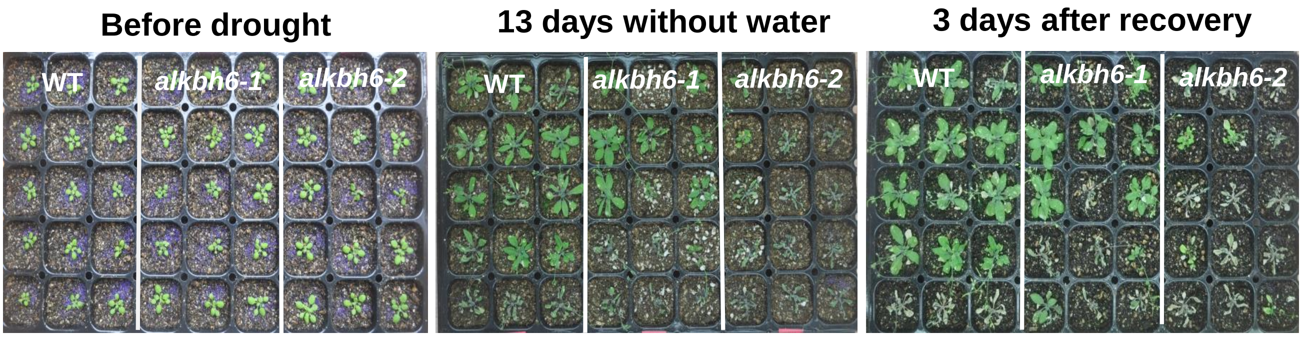

Supplemental Figure S6. Drought sensitivity of the *alkbh6* mutants. Twenty-days-old seedlings grown in soil were subjected to drought stress for 13 days, and survival rates were measured 3 days after recovery

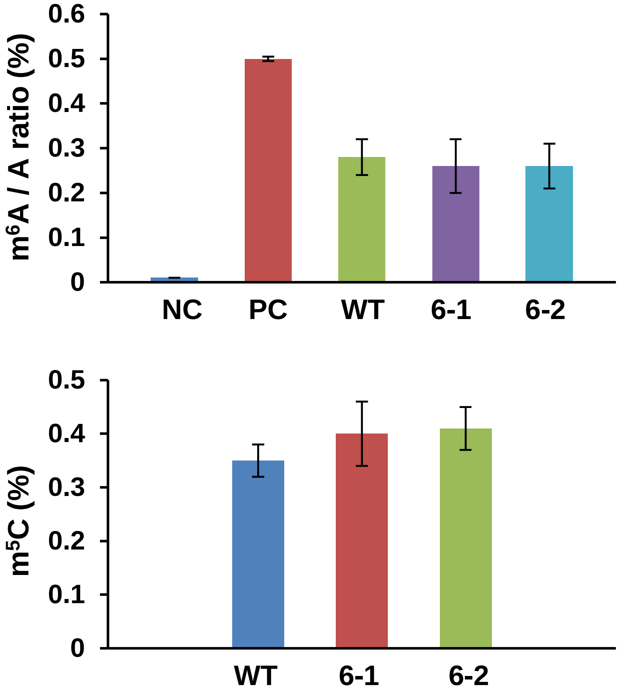

Supplementary Figure S7. Levels of m^{6}A/A and m^{5}C in the *alkbh6* mutants. Total RNA in 18-days-old wild type (WT) and *alkbh6* mutant (6-1 and 6-2) seedlings was extracted, and the levels of m^{6}A/A (%) and m^{5}C (%) were determined using an EpiQuik m^{6}A RNA Methylation Quantification Kit and a MethylFlash 5-mC RNA Methylation ELISA Easy Kit, respectively. Values are the mean ± SE obtained from three independent experiments. NC, negative control; PC, positive control.

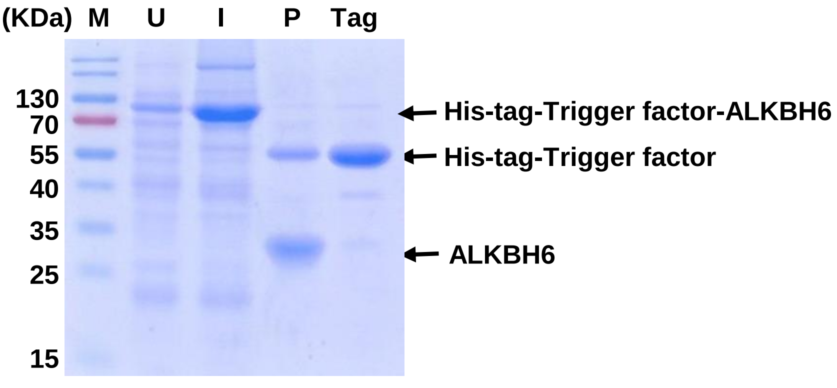

Supplementary Figure S8. Purification of the recombinant ALKBH6 protein. The His-tag-ALKBH6 fusion protein was expressed in *E. coli* and purified using a Ni-NTA His•Bind resin. M, molecular size marker; U, un-induced; I, induced; P, purified.
